# Predictor variables effect on the development of Burnout Syndrome in higher education professor

**DOI:** 10.1590/0034-7167-2024-0132

**Published:** 2024-09-20

**Authors:** Italo Everton Bezerra Barbosa, Aristéia Nunes Sampaio, Cristiane Pereira de Souza, Breno de Souza Mota, Carla Roberta Monteiro Miura, Cassiane Dezoti da Fonseca, Angélica Gonçalves Silva Belasco

**Affiliations:** I Universidade Federal de São Paulo. São Paulo, São Paulo, Brazil; II Universidade de São Paulo. São Paulo, São Paulo, Brazil

**Keywords:** Faculty, Anxiety, Quality of Life, Burnout, Psychological, Sleep Quality, Docentes, Ansiedad, Calidad de Vida, Agotamiento Psicológico, Calidad del Sueño

## Abstract

**Objectives:**

to correlate the development of Burnout Syndrome in higher education professors with the following variables: sociodemographic, economic, work, sleep pattern, level of anxiety and quality of life.

**Methods:**

observational, analytical, cross-sectional study with a quantitative approach. Data collection took place from August to November 2022, with 140 professors from a private higher education institution in the Western Brazilian Amazon.

**Results:**

professors who carried out activities outside institutional hours and who had sleep duration < 5 hours, presented lower scores in the personal fulfillment dimension of burnout, with (p=0.002) and (p=0.001), respectively. The higher the scores for the physical (p=0.001), psychological (p=0.000) and social relationships (p=0.002) domains of quality of life, the lower the personal fulfillment scores for the syndrome.

**Conclusions:**

through linear regression, it was evidenced that several variables explain the development of burnout. Institutional and governmental actions can minimize the negative influence of these variables.

## RESUMEN

**Objetivos:**

correlacionar variables sociodemográficas, económicas, laborales, patrón de sueño, nivel de ansiedad y calidad de vida con el desarrollo del Síndrome de Burnout en profesores de enseñanza superior.

**Métodos:**

es un estudio observacional, analítico y transversal con enfoque cuantitativo. Los datos se recogieron entre agosto y noviembre de 2022 de 140 profesores en una institución privada de enseñanza superior de la Amazonia Occidental brasileña.

**Resultados:**

los docentes que realizaban actividades fuera del horario institucional y que tenían una duración del sueño <5 horas presentaban puntuaciones más bajas en la dimensión de la realización personal del burnout, con (p=0,002) y (p=0,001), respectivamente. Cuanto mayores eran las puntuaciones de los dominios físico (p=0,001), psicológico (p=0,000) y relaciones sociales (p=0,002) de la calidad de vida, menores eran las puntuaciones de realización personal del síndrome.

**Conclusiones:**

la regresión lineal mostró que diversas variables explican el desarrollo del burnout. Las acciones institucionales y gubernamentales pueden minimizar la influencia negativa de dichas variables.

## INTRODUCTION

In the current social structure, the duties imposed on teachers go beyond the classroom, aiming to meet the educational objectives proposed by the higher education institution^(1)^. Regardless of the title, classes are supposed to remain updated and present high quality, incorporating greater parameters of productivity and work efficiency, to be able to teach classes to a large number of students, with all their needs and particularities^(2-3)^. This condition involves activities with intense interactions, which can produce results that affect not only the teaching-learning of students, but mainly the professional’s mental and physical health through work stress^(4-5)^.

The International Labor Organization (ILO) considers teaching work to be one of the most stressful, with many elements that cause various changes in Quality of Life (QoL)^(6)^. This process interacts with individual aspects and the work environment, since several factors corroborate this condition, such as the scarcity of material resources, carrying out administrative activities, participation in policies and institutional planning, favoring work overload and enabling a greater risk of illness through occupational stress^(7-8)^.

Teachers’ QoL can be influenced by various institutional aspects, given its subjective, complex and multidimensional nature and considering that the longer the working time, the less availability for personal and leisure activities^(9)^. Several conditions favor a decrease in QoL, predisposing the appearance of changes in sleep patterns, since all work generates exhausting factors, which are determining factors in the health-disease process^(10)^.

Changes in the quality, quantity and regularity of sleep are very harmful to health, generating reactions that change the individual’s physical, cognitive, occupational and social performance, as sleep is a periodic physiological phenomenon fundamental to human life^(11)^. In addition to these consequences, sleep-related disorders occur with great frequency today, with emphasis on insomnia, difficulty falling asleep, excessive daytime sleepiness, short sleep duration (less than 5 hours), early awakening and non-restorative sleep, which compromises the repair of brain energy metabolism^(12-13)^.

In this context, teaching can present professors with experiences in several situations that can generate anxiety through physical and mental exhaustion^(14)^. A situation that directly reflects on the development of work, making it difficult to carry out daily activities^(15)^. Anxiety is characterized as an emotion typical of human life, on the other hand, in its pathological condition, it manifests itself intensely with symptoms that can generate suffering, damaging the individual’s psychic and somatic functioning^(16)^.

Numerous sociodemographic and work variables aggravate stressful responses and compromise patterns of physiological responses caused by negative experiences in the work sector^(17-18)^. This condition favors an intense imbalance in the body, predisposing to the appearance of Burnout Syndrome (BS), resulting from chronic interpersonal stressors resulting from work and which can affect the body, occurring mainly in workers who have direct and/or indirect contact with other people^(19)^.

The word burnout was first described in 1974, by the psychoanalyst Freudenberger^(20)^. It is a symptomatological condition characterized by three interdependent dimensions: emotional exhaustion, which corresponds to emotional and physical exhaustion caused by work overload; depersonalization: which refers to emotional imbalance and favors cold and impersonal relationships with the individuals who make up the work environment and, finally; personal fulfillment, which corresponds to feelings of unproductivity at work through negative self-evaluation^(21)^.

Several factors can favor the appearance of burnout in teachers, causing complications that can significantly interfere with the educational system. The chronicity of stress, generated by the overload experienced at work, compromises work performance, presenting negative effects that are highly harmful to the worker’s health^(15)^. In Brazil, countless workers feel unable to carry out their activities. Around 72% of the economically active population has a high level of stress and at least 30% of these can be classified with BS^(10-20)^. Identifying which variables favor the development of BS enables the implementation of measures capable of minimizing or banishing the chances of manifestation of this syndrome and can also contribute to the search for improving the QoL of these professionals.

## OBJECTIVES

To correlate the development of Burnout Syndrome in higher education professors with the following variables: sociodemographic, economic, work, sleep pattern, level of anxiety and quality of life.

## METHODS

### Ethical aspects

Considering the ethical aspects of scientific research, this study was submitted to the Research Ethics Committee of the Federal University of São Paulo (UNIFESP), in accordance with Resolution 466/2012 of the National Health Council. It is important to note that all participants read, signed and agreed to the Free and Informed Consent Form.

### Study design, period and location

Observational, analytical, cross-sectional research with a quantitative method, developed in accordance with the Strengthening the Reporting of Observational Studies in Epidemiology (STROBE) tool. Held at a private higher education institution in the Western Brazilian Amazon. Data collection was carried out from August to November 2022.

### Sample, inclusion and exclusion criteria

The study sample was defined according to the number of permanent and contracted teachers in all shifts. According to the institution where the research was carried out, the total number of teachers was 210, of which 173 met the inclusion criteria, which is the population considered for calculating the final study sample. Aiming to identify possible participants, the sample was calculated using the formula n = [EDFF*Np(1-p)]/[(d 2 /Z 2 1-α/2 *(N-1)+p*(1 -p)], through the Open Epi platform, adopting a maximum sample estimate error of 5% and a confidence level of 95%. The sample initially consisted of 120 teachers, taking into account possible losses and refusals, the sample was increased by 20%, resulting in 144. The interviews lasted between 40 and 50 minutes and were carried out during breaks or after classes.

The inclusion criteria were: being over 18 years old, being a teacher at the institution for at least one year and working directly in the classroom with students. The non-inclusion criteria were: being absent due to absence or vacation during the period of data collection and working only in managerial and/or administrative activities.

### Study protocol

Firstly, contact was made with the institution’s management to request authorization to carry out the study. After approval, a time was scheduled with the course coordinators in order to obtain a list of teachers and working hours, then it was The invitation was made to each teacher, personally, through an individual explanation about the importance and purpose of the research, if they agreed to participate, the signature of the Consent form was collected, followed by the application of the research instruments.

### Data collection instruments

The sociodemographic variables included in the questionnaire were: sex, age, nationality, marital status, number of children, skin color and education. In terms of work: length of time at the institution, number of students for guidance and per class, workload, number of employment relationships and salary range.

To evaluate the teachers’ QoL, the translated and validated version for Brazil of the World Health Organization Quality of Life Assessment (WHOQOL - bref) instrument, developed by the WHO, was used, consisting of 26 questions, two of which were about the quality of general life (perception of QoL and satisfaction with health) and the others subdivided into four domains: physical, psychological, social relationships and environment. The questions are of the Likert type (1 to 5 points), with scores ranging from 0 to 100 and, the higher the score, the better the QoL^(22)^.

The teachers’ sleep quality was verified using the Pittsburgh Sleep Quality Index Questionnaire (PSQI), translated and validated in Brazil, consisting of 19 questions that evaluate seven components that involve the sleep pattern: quality, latency, duration, efficiency, disorders, medication and dysfunction. Each component receives a score that varies from 0 to 3, so that the instrument’s final score can vary from 0 to 21. Scores greater than 5 imply poor sleep quality and greater than or equal to 10 indicate sleep disturbance^(23)^.

To assess the level of anxiety, the State-Trait Anxiety Inventory (STAI), translated and adapted for Brazil, was used. It is used to identify the level of anxiety based on two factors: state, how the person feels at the “moment” and the trait, how they “generally feel”. The STAI is a 20-question self-administered questionnaire composed of two distinct scales. The interpretation of the results is based on the sum of responses ranging from 1 to 4, the higher the score the greater the intensity of anxiety symptoms, being divided into three categories: low (20 to 40); medium (41 to 60) and high (61 to 80)^(24)^.

The Maslach Burnout Inventory (MBI) was used to assess the occurrence of Burnout Syndrome. Consisting of 22 items distributed across three dimensions: emotional exhaustion (9 items), depersonalization (5 items) and personal fulfillment (8 items). The instrument uses a Likert-type scale, ranging from 0 to 6. The presence of three dimensions indicates the manifestation of BS and the presence of two indicates a high risk for its development^(25)^.

### Analysis of results and statistics

The research data was stored in a secure location and only the researchers had access. All participants received an identification number to ensure anonymity and confidentiality of information.

After data collection, the results were grouped, tabulated and organized in an electronic database by typing them into a spreadsheet in the Microsoft Excel^®^ application. The Statistical Package for the Social Sciences (SPSS) version 20.0^®^ for Windows was used for data analysis.

The frequency and percentage were calculated in the descriptive analysis of the categorical variables. Spearman’s Correlation Coefficient was used to correlate the MBI dimensions with WHOQOL-Bref domains and the time dedicated to work outside the institution. The Chi-square test was used to compare the components and classify the sleep scale according to salary range and number of jobs. The Mann-Whitney test (2 categories) and Kruskal-Wallis test (3 or more categories) were used to compare the MBI dimensions with Sleep Quality (Components and Classification), STAI (State and Trait), sociodemographic and work variables.

To verify which independent variables (WHOQOL-Bref, IDATE, PSQI, education, time at the institution, number of students per class and for guidance, number and employment relationship, salary range, time dedicated to teaching work outside the institution) best explained the dimensions of the dependent variable (Burnout), Multiple Linear Regression was used. A significance level of 5% was adopted (p-value < 0.05)^(26)^.

## RESULTS

Of the 144 selected initially, 23 were absent during collection and six refused to participate. However, in order to reach a sample number close to that expected, 25 new collections were carried out with participants who met the inclusion criteria and had not yet been included, resulting in a final sample composed of 140 teachers, with acceptance and participation from eight different courses: Dentistry (5.7%); Environmental Engineering (10%); Nutrition (6.4%); Psychology (7.1%); Biomedicine (8.6%); Physiotherapy (8.6%); Pharmacy (7.9%); Physical Education (7.1%); Administration (9.3%); Veterinary Medicine (18.6%) and Nursing (10.7%).

The average age was 38.5 (+7.3) years. There was a predominance of women (54.3%), aged between 26 and 40 years (58.6%), married marital status (47.1%), with one child (38.6%), and self-declared skin white (57.9%). Regarding work characteristics, they were hourly workers (90.7%), specialists (48.6%), with two or more employment contracts (52.1%), salary range of up to R$5,000.00 (85.7%), with more than 40 students per class (75.7%), with up to 5 years at the institution (77.9%) and up to six students for supervision of course completion work (87.9%).

The analysis of the WHOQOL-Bref demonstrated that the teachers’ perception of QoL was predominantly “neither bad nor good” (54.3%) and that they were “dissatisfied with their health” (41.4%). Table 1 demonstrates the correlation between the WHOQOL-Bref domains and the MBI dimensions.

**Table 1:** Correlation of World Health Organization Quality of Life Assessment domains by Maslach Burnout Inventory dimensions of higher education teachers, Manaus, Amazonas, Brazil, 2022, N=140

WHOQOL-Bref		Maslach Burnout Inventory		
		Emotional Exhaustion	Depersonalization	Personal fulfillment
Physical	R	-0.13	-0.04	-0.27
	*p* value	0.114	0.607	0.001
Psycological	R	-0.22	-0.02	-0.48
	*p* value	0.010	0.858	0.000
Social Relations	R	-0.10	-0.19	-0.31
	*p* value	0.256	0.024	0.002
Environment	R	-0.07	-0.24	-0.27
	*p* value	0.420	0.003	0.001

*WHOQOL-Bref - World Health Organization Quality of Life Assessment; R - Spearman Correlation Coefficient*

A significant correlation was found between the variable “how much time the teacher has available to dedicate to work through activities outside institutional hours” (correcting tests/assignments, class preparation and guidance) and the personal achievement dimension of the MBI (p=0,0023).

The overall PSQI score showed a predominance in the classification of poor sleep (66.4%). Of these, 24 teachers (17.1%) achieved scores that classified them as having a sleep disorder. The association of this instrument with the emotional exhaustion domain of the MBI showed significant results (Table 2).

**Table 2:** Association between the Pittsburgh Sleep Quality Index Questionnaire and the Emotional Exhaustion dimension of the MBI of higher education teachers, Manaus, Amazonas, Brazil, 2022, N=140

Components - PSQI	Emotional Exhaustion				*p* value
	n (140)	Mean (SD)	Median	Minimum-Maximum	
Subjective sleep quality					
Very good	22	32.7 (6.01)	34	19-41	0.005^**^
Good	70	31.1 (5.78)	31	11-47	
Bad/Really bad	48	34.1 (5.27)	34	22-49	
Sleep disturbance (times/week)					
1 to 2	99	31.6 (5.61)	32	11-47	0.029^*^
≥ 3	41	34.3 (5.78)	34	22-49	
Use of sleeping medication (times/week)					
None	82	30.9 (5.84)	31	11-46	0.001^**^
< 1	25	34.7 (5.3)	34	28-49	
1 a 2	33	34.4 (4.84)	34	22-44	
Dysfunction during the day					
None	13	32.7 (7.34)	30	26-49	0.005^**^
Small	89	31.5 (5.64)	32	11-47	
Moderate/A lot	38	34.4 (5.07)	35	22-44	
Pontuação Global					
Good	23	30.1 (7.14)	30	11-42	0.007^**^
Bad	93	32.4 (5.51)	32	15-49	
Presence of sleep disorder	24	34.9 (4.35)	35.5	25-44	

PSQI - Pittsburgh Sleep Quality Index Questionnaire;

* Mann-Whitney test;

** Kruskal-Wallis test.

Participants with poor/very poor subjective sleep quality (p=0.023) and daytime dysfunction (p=0.013) had a higher score in the depersonalization dimension of the MBI. Teachers with sleep duration < 5 hours had a higher score in the dimension of personal fulfillment (p=0.001). Individuals with a sleep disorder ≥ 3 times a week had a higher score in that MBI dimension (p=0.010).

The association of state and trait STAI with personal MBI achievement showed significant results (Table 3).

**Table 3:** Association between the State-Trait Anxiety Inventory and the Maslach Burnout Inventory dimensions of higher education teachers, Manaus, Amazonas, Brazil, 2022, N=140

STAI	Depersonalization				*p* value^*^
	n	Mean (SD)	Median	Minimum-Maximum	
State					
Low	34	17.6 (3.16)	18	6-24	0.188^*^
Medium	96	17.1 (2.67)	17	10-23	
High	10	18.3 (2.63)	18	13-23	
Trait					
Low	22	18.7 (2.41)	19	15-24	0.034^*^
Medium	113	17.1 (2.82)	17	6-23	
High	5	17.4 (2.07)	17	15-20	
	**Personal fulfillment**				
State					
Low	34	19.4 (5.23)	20.5	6-28	0.006^*^
Medium	96	22.5 (4.53)	23	7-32	
High	10	21.1 (3.77)	20.5	13-26	
Trait					
Low	22	17.4 (5.58)	16.5	6-27	0.002^*^
Medium	113	22.4 (4.3)	23	7-32	
High	5	21.4 (2.97)	21	18-26	

STAI - State-Trait Anxiety Inventory;

* Kruskal-Wallis test.

The variables number of students per room and for guidance showed significance in association with the emotional exhaustion dimension of the MBI (Table 4). Teachers with more than 40 students per room had a higher score in the depersonalization dimension than participants with up to 40 students per room (p=0.006). Participants with more than five years at the institution had a higher score in that dimension (p=0.032). Individuals with up to two students for guidance had a higher score in the dimension of personal achievement (p=0.045).

**Table 4:** Association between work variables and the emotional exhaustion dimension of the Maslach Burnout Inventory of higher education teachers, Manaus, Amazonas, Brazil, 2022, N=140

	Emotional Exhaustion				*p* value^*^
	n	Mean (SD)	Median	Minimum-Maximum	
Number of students per room					
Until 40	34	30.62 (5.74)	30.5	15-46	0.017^*^
More than 40 students	106	33 (5.69)	33	11-49	
Number of students for guidance					
Until 2	89	31.82 (5.07)	32	11-46	
2 to 4	23	36 (5.84)	36	27-49	0.028^**^
4 to 6	11	31.18 (5.46)	33	21-38	
More than 6	17	31.53 (7.76)	34	15-44	

* Mann-Whitney test;

** Kruskal-Wallis test.

Multiple Linear Regression presented the predictor variables that best explained the burnout dimension scores (Table 5).

**Table 5:** Predictive variables of the Maslach Burnout Inventory dimensions of higher education teachers, Manaus, Amazonas, Brazil, 2022, N=140

Dimensions	Multiple Linear Regression		*p* value^*^
	Variables	Estimates	
Emotional Exhaustion	Constant	38,58	<0,001
	2 to 4 students for guidance	3,44	0,004
	WHOQOL - Physical Domain	-0,14	0,001
	Poor subjective sleep quality	-2,13	0,022
	More than 40 students per class	2,10	0,049
Depersonalization	Constant	16,76	<0,001
	More than 40 students per class	1,87	0,003
	Lots of sleep dysfunction during the day	1,42	0,004
	STAI Trait - Medium	-1,58	0,004
Personal fulfillment	Constant	32,94	<0,001
	WHOQOL - Psychological Domain	-0,12	0,001
	Long teaching hours outside the institution	-0,21	0,002
	Presence of sleep disturbance in the global score	2,02	0,034
	WHOQOL - Social Relations Domain	-0,07	0,004
	Use of sleeping medication - ≥ 3 times a week	1,72	0,038

* *Significance level of 5% (p-value < 0.05); WHOQOL - World Health Organization Quality of Life Assessment; STAI - State-Trait Anxiety Inventory.*

## DISCUSSION

QoL is a frequent focus of scientific studies, as its scores can act as subjective indicators of health status^(27)^. The results of this study indicate that the higher the score in the psychological domain of the WHOQOL-Bref, the lower the score in the emotional exhaustion dimension of the MBI. Furthermore, higher scores in the environment and social relationships domains correspond to lower depersonalization scores. This phenomenon can be attributed to the intrinsic characteristics of the profession, which is influenced by several elements that contribute to professional burnout, including, but not limited to: excessive crowding in rooms, insufficient remuneration, lack of security and little or no autonomy^(28-29)^.

The higher the score in the environment, social relationships, psychological and physical domain, the lower the score in the personal fulfillment dimension in this research. The understanding of QoL is individual, involving objective/subjective parameters correlated with numerous variables, interconnected with health, leisure, education, environment, safety, urban planning and technology^(30)^. In BS, this dimension is classified in reverse, the lower the score, the greater the severity. In view of this, due to their dissatisfaction, teachers may present conditions that harm their performance, causing feelings of hopelessness, tension, loneliness, weakness, worry and apathy towards students and co-workers^(31)^.

Carrying out work activities outside institutional hours can lead to numerous harms to the teacher’s emotional health^(32)^. A situation that can result in the appearance of sleep disorders, since only (20%) of the teachers in this study reported sleeping more than seven hours, with sleep latency of up to one hour (43.6%). A condition that can be explained by the dynamics of the profession, marked by extra-class activities, such as correcting tests, preparing classes, advising course completion work and participating in research and extension activities^(33)^.

Poor sleep quality is described as common among higher education teachers^(34)^. Other studies that used the same instrument presented lower percentages, which favored harm to the professional’s biopsychosocial health^(35-36)^. Increased work overload corroborates the manifestation of sleep disorders, a work condition that explains the poor quality of sleep in this and other investigations^(37)^. Teachers with excessive daytime sleepiness can develop metabolic, physical and endocrine disorders, predisposing to gastric and intestinal problems, immune system deficits and eating disorders, affecting QoL^(38)^.

Studies highlight that the use of medication is frequent among teachers. A survey showed that 21.9% of teachers used medications (prescribed or not by the doctor), such as anxiolytics, hypnotics, analgesics and antidepressants^(33,39)^. Self-medication is most often carried out as a way to alleviate symptoms triggered by work. However, incorrect use can have several health consequences, with a focus on dependence, intoxication and resistance to medicines^(40)^.

Teachers with sleep duration < 5 hours per night may experience greater cognitive decline over time. A situation that can be explained by high demands at work, having little time to carry out personal activities, triggering signs and symptoms correlated with BS^(41)^. These work conditions interfere with QoL and the work environment, presenting several losses in class planning. Factor demonstrated as a greater risk of illness due to occupational stress in another investigation^(42)^.

Anxiety is among the symptoms that appear most in research carried out with higher education teachers and affects younger individuals, under the age of 40, possibly because they are at the beginning of their careers, have little experience and professional maturity, which makes them more susceptible to work frustrations^(16)^. This corroborates the appearance of psychosomatic symptoms, such as stress, insomnia, Systemic Arterial Hypertension (SAH) and this work situation favors the development of BS^(43)^.

In relation to sociodemographic and labor variables, studies have shown that the longer the professional training period, the greater the negative evaluation at work^(44-45)^. And they highlighted that single people are at greater risk of developing burnout, as they dedicate themselves, most of the time, only to work. In this context, the existence of an emotional partner is considered a protective factor, providing greater security, support and encouragement to deal with work-related events^(41)^.

Working in two or more educational institutions can be related to financial, personal and working conditions^(46)^. An international study carried out in Pakistan demonstrated that 70.3% of teachers showed moderate emotional exhaustion and severe personal fulfillment^(47)^. This condition is associated with problems of personal identity and lack of recognition in the work sector, favoring professional burnout^(48)^.

The high number of students per class increases work overload, as it intensifies stress, predisposing it to chronicity. This condition causes harm to the psychological and physical health of teachers, predisposing to the appearance of dyspnea, nausea, tremors and tingling in the hands, tachycardia, low productivity, decreased sexual interest, lack of appetite, excessive sweating, myalgia and difficulty falling asleep^(49)^. Added to this, professionals who have been at the educational institution for longer may present signs and symptoms correlated with BS, with a focus on absenteeism^(17-18)^.

The predictive variables that correlated with professional burnout, in this study, require serious evaluation and analysis so that preventive measures can be instituted to prevent the development of BS. Instructing teachers on strategies capable of reducing the consequences caused by work overload becomes a fundamental measure capable of having a positive impact on QoL and reducing the possibilities of sleep-related disorders. Including the practice of physical and leisure activities in your routine can help reduce the level of anxiety caused by the work environment, reducing the chances of burnout^(50)^.

### Study limitations

This research was carried out in a private higher education institution, therefore, its results cannot be generalized. However, comparative studies in public and private institutions must be considered, aiming to analyze similar or divergent data, given different working conditions.

### Contributions to practice

This research minimizes the gap in the scientific literature on the topic addressed, highlighting a series of modifiable variables that support the development of BS in the workplace.

## CONCLUSIONS

In the present study, several predictive variables were identified that favored psychological exhaustion in the teaching class. When considering the proportion of BS, through linear regression, it was evident that the following variables best explain its development: poor sleep quality, more than 40 students per class, a lot of sleep dysfunction during the day, STAI-Trait, WHOQOL in the Psychological and Social Relationships domains, long teaching hours outside the institution, presence of sleep disorders in the global score, use of sleeping medication (≥ 3 times a week) and students for guidance. Therefore, it is concluded that the educational system contributes to the worsening of this situation, since the dynamics of the profession are marked by numerous activities that are carried out outside institutional hours, predisposing to anxiety attacks, changes in QoL and sleep patterns. , causing harm to the professional’s biopsychosocial health.

Given the above, it is possible to reflect the current situation of teachers at the private higher education institution, in focus, highlighting the need for alertness in the management of the predictive variables that favored the physical and emotional exhaustion of the evaluated professionals.

Developing health promotion and prevention actions within the university context is essential to avoid compromising the QoL, sleep and health of teachers with consequent compromising of teaching and learning for students. However, new studies, actions and programs that promote the exclusion of these conditions that correlate with the development of BS are necessary.
