# Supplementary material for: Predictor variables effect on the development of Burnout Syndrome in higher education professor
Source: Rev Bras Enferm. 2024 Sep 20;77(4):e20240132. doi: 10.1590/0034-7167-2024-0132 (PMC11419690; doi:10.1590/0034-7167-2024-0132)
Supplement: Translation

# Translation: Efeito das variáveis preditoras no desenvolvimento da Síndrome de *Burnout* em docentes do ensino superior

**DOI:** 10.1590/0034-7167-2024-0132pt

**Authors:** Italo Everton Bezerra Barbosa, Aristéia Nunes Sampaio, Cristiane Pereira de Souza, Breno de Souza Mota, Carla Roberta Monteiro Miura, Cassiane Dezoti da Fonseca, Angélica Gonçalves Silva Belasco

**Affiliations:** I Universidade Federal de São Paulo. São Paulo, São Paulo, Brasil; II Universidade de São Paulo. São Paulo, São Paulo, Brasil

**Keywords:** Docentes, Ansiedade, Qualidade de Vida, Esgotamento Psicológico, Qualidade do Sono.

## Abstract

**Objetivos:**

correlacionar as variáveis sociodemográficas, econômicas, de trabalho, padrão do sono, nível de ansiedade e qualidade de vida ao desenvolvimento da Síndrome de *Burnout* em docentes do ensino superior.

**Métodos:**

estudo observacional, analítico, transversal e com abordagem quantitativa. A coleta dos dados ocorreu de agosto a novembro de 2022, com 140 docentes de uma instituição de ensino superior privada, da Amazônia Ocidental Brasileira.

**Resultados:**

docentes que realizavam atividades fora do horário institucional e que tinham duração do sono < 5 horas, apresentaram menores escores na dimensão da realização pessoal do *burnout*, com (p=0,002) e (p=0,001), respectivamente. Quanto maior os escores dos domínios físico (p=0,001), psicológico (p=0,000) e relações sociais (p=0,002) da qualidade de vida, menores foram os escores de realização pessoal da síndrome.

**Conclusões:**

por meio da regressão linear, evidenciou-se que diversas variáveis explicam o desenvolvimento do *burnout*. Ações institucionais e governamentais podem minimizar a influência negativa dessas variáveis.

## INTRODUÇÃO

Na estrutura social vigente, as atribuições impostas aos docentes vão além da sala de aula, visando atender aos objetivos educacionais propostos pela instituição de ensino superior^(1)^. Independente da titulação, a classe é instruída a manter-se atualizada e qualificar-se sempre, incorporando maiores parâmetros de produtividade e eficiência laboral, para estar apta a ministrar aulas para um elevado número de alunos, com todas as suas necessidades e particularidades^(2-3)^. Esta condição envolve atividades com intensas interações, podendo produzir resultados que afetam não apenas o ensino-aprendizagem dos alunos, mas principalmente a saúde mental e física do profissional mediante ao estresse laboral^(4-5)^.

A Organização Internacional do Trabalho (OIT) considera o ofício docente como um dos mais estressantes, com muitos elementos que causam várias mudanças na Qualidade de Vida (QV)^(6)^. Esse processo interage com aspectos individuais e com o ambiente de trabalho, uma vez que diversos fatores corroboram para essa condição, tais como a escassez de recursos de materiais, a realização de atividades administrativas, além da participação nas políticas e no planejamento institucional, favorecendo a sobrecarga de trabalho e viabilizando maior risco de adoecimento por intermédio do estresse ocupacional^(7-8)^.

Sendo assim, a QV do docente pode sofrer influência de diversos aspectos institucionais, tendo em vista o seu caráter subjetivo, complexo e multidimensional e considerando que quanto maior o tempo de trabalho, menor é a disponibilidade para as atividades pessoais e de lazer^(9)^. Logo, diversas condições favorecem a diminuição da QV, predispondo o aparecimento de alterações no padrão do sono, uma vez que, todo trabalho é gerador de fatores desgastantes, que são determinantes no processo saúde-doença^(10)^.

Além do mais, mudanças na qualidade, quantidade e regularidade do sono são muito danosas à saúde, gerando reações que mudam o desempenho físico, cognitivo, ocupacional e social do indivíduo, pois o sono é um fenômeno fisiológico periódico fundamental para a vida humana^(11)^. Além dessas consequências, distúrbios relacionados ao sono ocorrem com grande frequência na atualidade, com destaque para a insônia, dificuldade em adormecer, sonolência diurna excessiva, sono de curta duração (inferior a 5 horas), despertar precoce e sono não restaurador, o que compromete a reparação do metabolismo energético cerebral^(12-13)^.

Neste contexto, a classe docente vivencia diversas situações que podem gerar quadros de ansiedade por intermédio da exaustão física e mental^(14)^. Situação que reflete diretamente no desenvolvimento do trabalho, fazendo com que haja dificuldade no cumprimento das atividades cotidianas^(15)^. A ansiedade é caracterizada como uma emoção própria da vida humana, por outro lado, em sua condição patológica, manifesta-se de forma intensa e com sintomas que podem gerar sofrimento, prejudicando o funcionamento psíquico e somático do indivíduo^(16)^.

Consequentemente, inúmeras variáveis sociodemográficas e laborais agravam respostas estressoras e comprometem padrões de respostas fisiológicas ocasionadas por experiências negativas vivenciadas no setor de trabalho^(17-18)^. Essa condição favorece um desequilíbrio intenso no organismo, predispondo ao aparecimento da Síndrome de *Burnout* (SB), resultante de estressores interpessoais crônicos decorrentes do trabalho e que podem afetar o organismo, ocorrendo principalmente em trabalhadores que possuem contato direto e/ou indireto com outras pessoas^(19)^.

Porquanto, a palavra *burnout* foi descrita pela primeira vez em 1974, pelo médico psicanalista Freudenberger^(20)^. É um quadro sintomatológico caracterizado por três dimensões interdependentes: exaustão emocional, que corresponde ao esgotamento emocional e físico concebido pela sobrecarga de trabalho; despersonalização, que se refere ao desequilíbrio emocional e favorece o relacionamento frio e impessoal com os indivíduos que compõem o ambiente de trabalho; e, por fim, a realização pessoal, que corresponde a sentimentos de improdutividade no trabalho por meio da autoavaliação negativa^(21)^.

Diversos fatores podem favorecer o aparecimento de *burnout* nos professores, acarretando complicações capazes de interferir significativamente no sistema educacional. A cronicidade do estresse, gerado pela sobrecarga vivenciada no trabalho, compromete o desempenho laboral, apresentando efeitos negativos que são altamente prejudiciais à saúde do trabalhador^(15)^. No Brasil, inúmeros trabalhadores sentem-se incapacitados para exercerem suas atividades. Cerca de 72% da população economicamente ativa apresenta elevado índice de estresse, e pelo menos 30% desses podem ser enquadrados na SB^(10-20)^. Identificar quais variáveis favorecem o desenvolvimento da SB possibilita a implantação de medidas capazes de minimizar ou banir as chances de manifestação da referida síndrome e pode ainda contribuir na busca de melhoria da QV desses profissionais.

## OBJETIVOS

Correlacionar as variáveis sociodemográficas, econômicas, de trabalho, padrão do sono, nível de ansiedade e qualidade de vida ao desenvolvimento da Síndrome de *Burnout* em docentes do ensino superior.

## MÉTODOS

### Aspectos éticos

Considerando os aspectos éticos da pesquisa científica, este estudo foi submetido ao Comitê de Ética em Pesquisa da Universidade Federal de São Paulo (UNIFESP), em conformidade com a Resolução 466/2012, do Conselho Nacional de Saúde (CNS). Ressalta-se que todos os participantes leram, concordaram e assinaram o Termo de Consentimento Livre e Esclarecido (TCLE).

### Desenho, período e local do estudo

Pesquisa observacional, analítica, de corte transversal e com método quantitativo, desenvolvida de acordo com a ferramenta *Strengthening the Reporting of Observational Studies in Epidemiology* (STROBE) e realizada em uma instituição de ensino superior privada, da Amazônia Ocidental Brasileira. A coleta dos dados foi realizada de agosto a novembro de 2022.

### Amostra, critérios de inclusão e de exclusão

A amostra do estudo foi definida conforme a quantidade de docentes efetivos e contratados em todos os turnos. Segundo a instituição de ensino onde foi realizada a pesquisa, o número total de docentes era de 210, dos quais 173 atendiam aos critérios de inclusão, sendo essa a população considerada para o cálculo da amostra final do estudo. Visando identificar os possíveis participantes, foi calculada a amostra por meio da fórmula n = [EDFF*Np(1-p)]/[(d 2 /Z 2 1-α/2 *(N-1)+p*(1-p)], através da plataforma Open Epi, adotando um erro máximo da estimativa amostral de 5% e um nível de confiança de 95%. Sendo assim, a amostra inicialmente foi composta por 120 docentes. Ao levar em consideração as possíveis perdas e recusas, aumentou-se a amostra em 20%, resultando em 144. Além do mais, as entrevistas duraram entre 40 e 50 minutos e foram realizadas durante o intervalo ou após as aulas.

Os critérios de inclusão foram: idade superior a 18 anos, ser docente na instituição por no mínimo um ano e atuar diretamente em sala de aula com os alunos. Os critérios de não inclusão foram: estar ausente por afastamento ou férias durante o período da coleta de dados e atuar somente em atividades gerenciais e/ou administrativas.

### Protocolo do estudo

No primeiro momento, ocorreu o contato com a direção da instituição para a solicitação da autorização para a realização do estudo. Após a aprovação, foi agendado horário com as coordenações de curso, visando conseguir a lista de docentes e os horários de trabalho. Em seguida, foi feito o convite pessoalmente a cada professor, por meio de explanação individual sobre a importância e a finalidade da pesquisa. Caso aceitassem participar, era colhida a assinatura do TCLE e seguida da aplicação dos instrumentos de pesquisa.

### Instrumentos de coleta de dados

As variáveis sociodemográficas inseridas no questionário foram: sexo, idade, nacionalidade, estado civil, número de filhos, cor da pele e escolaridade. No aspecto laboral: tempo de instituição, quantidade de alunos para orientação e por sala, carga horária, quantidade de vínculos empregatícios e faixa salarial.

Para avaliar a QV dos docentes, foi utilizada a versão traduzida e validada para o Brasil do instrumento *World Health Organization Quality of Life Assessment* (WHOQOL - bref), que foi desenvolvido pela OMS e composto por 26 questões, dentre as quais duas eram sobre a qualidade de vida geral (percepção da QV e satisfação com a saúde) e as demais subdivididas em quatro domínios: físico, psicológico, relações sociais e meio ambiente. As perguntas eram do tipo Likert (1 a 5 pontos), com pontuações que variam de 0 a 100 e, quanto maior a pontuação, melhor a QV^(22)^.

Já a qualidade do sono dos docentes foi verificada por meio do Questionário Índice de Qualidade do Sono de Pittsburgh (PSQI), traduzido e validado no Brasil, composto por 19 questões que avaliam sete componentes que envolvem o padrão do sono, sendo: qualidade, latência, duração, eficiência, distúrbios, medicação e disfunção. Cada componente recebe uma pontuação que varia de 0 a 3, de forma que a pontuação final do instrumento pode variar de 0 a 21. Escores maiores que 5 implicam na qualidade do sono ruim e maiores ou iguais a 10 indicam distúrbio do sono^(23)^.

Para o julgamento do nível de ansiedade, foi empregado o Inventário de Ansiedade Traço-Estado (IDATE), traduzido e adaptado para o Brasil, e que é utilizado para identificar o nível de ansiedade com base em dois fatores: o estado, como a pessoa se sente no “momento”; e o traço, como ela “geralmente se sente”. O IDATE é um questionário autoaplicável de 20 questões composto por duas escalas distintas. Ademais, a interpretação dos resultados baseia-se na somatória das respostas que variam de 1 a 4, quanto maior o escore, maior a intensidade dos sintomas de ansiedade, sendo dividido em três categorias: baixo (20 a 40), médio (41 a 60) e alto (61 a 80)^(24)^.

Por último, foi utilizado o *Maslach Burnout Inventory* (MBI) para avaliar a ocorrência da Síndrome de *Burnout*, E que é composto por 22 itens distribuídos em três dimensões: exaustão emocional (9 itens), despersonalização (5 itens) e realização pessoal (8 itens). O instrumento utiliza a escala do tipo Likert, com variação de 0 a 6. A presença das três dimensões indica a manifestação da SB e a presença de duas, alto risco para o desenvolvimento dela^(25)^.

### Análise dos resultados e estatística

Os dados da pesquisa foram armazenados em local seguro e somente os pesquisadores tinham acesso. Ademais, todos os participantes receberam um número de identificação para assegurar o anonimato e a confidencialidade das informações. Após a coleta de dados, os resultados foram agrupados, tabulados e organizados em um banco de dados eletrônico por meio de digitação em planilha, no aplicativo *Microsoft Excel* ^®^. Para a análise dos dados foi utilizado o programa *Statistical Package for the Social Sciences* (SPSS) versão 20.0^®^ *for Windows*.

Com efeito, calculou-se a frequência e o percentual na análise descritiva das variáveis categóricas. Foi utilizado o Coeficiente de Correlação de Spearman, para correlacionar as dimensões do MBI com domínios WHOQOL-Bref e o tempo que se dedica ao trabalho fora da instituição. Ainda, foi utilizado o teste Qui-quadrado para comparar os componentes e para classificar a escala do sono de acordo com a faixa salarial e a quantidade de empregos. Já os testes de Mann-Whitney (2 categorias) e Kruskal-Wallis (3 ou mais categorias) foram utilizados para comparar as dimensões do MBI com a Qualidade do Sono (Componentes e Classificação), IDATE (Estado e Traço), variáveis sociodemográficas e laborais.

Por fim, para verificar quais variáveis independentes (WHOQOL-Bref, IDATE, PSQI, escolaridade, tempo de instituição, quantidade de alunos por sala e para orientação, quantidade e vínculo empregatício, faixa salarial, tempo que se dedica ao trabalho docente fora da instituição) melhor explicaram as dimensões da variável dependente (*Burnout*), foi utilizada a Regressão Linear Múltipla. Além do mais, adotou-se um nível de significância de 5% (p-valor < 0,05)^(26)^.

## RESULTADOS

Dos 144 docentes selecionados no primeiro momento, 23 estavam ausentes durante a coleta e seis se recusaram a participar. Contudo, de modo a alcançar um número amostral próximo ao esperado, realizou-se 25 novas coletas com participantes que se enquadravam nos critérios de inclusão e ainda não tinham sido incluídos. Ao final, resultou em uma amostra final composta por 140 docentes, com a aceitação e participação de oito diferentes cursos: Odontologia (5,7%), Engenharia Ambiental (10%), Nutrição (6,4%), Psicologia (7,1%), Biomedicina (8,6%), Fisioterapia (8,6%), Farmácia (7,9%), Educação Física (7,1%), Administração (9,3%), Medicina Veterinária (18,6%) e Enfermagem (10,7%).

Assim, a média de idade foi de 38,5 (+7,3) anos. Houve predomínio de mulheres (54,3%), com faixa etária entre 26 e 40 anos (58,6%), estado civil casada (47,1%), com um filho (38,6%) e autodeclarados de pele branca (57,9%). Quanto às características laborais, eram horistas (90,7%), especialistas (48,6%), com dois ou mais vínculos empregatícios (52,1%), faixa salarial de até R$ 5.000,00 (85,7%), possuindo mais de 40 alunos por sala (75,7%), com até 5 anos de instituição (77,9%) e até seis alunos para orientação de trabalhos de conclusão de curso (87,9%).

Em vista disso, a análise do WHOQOL-Bref demonstrou que a percepção da QV dos docentes obteve predomínio de “nem ruim nem boa” (54,3%) e apresentavam-se “insatisfeitos com a saúde” (41,4%). A Tabela 1 demonstra a correlação entre os domínios do WHOQOL-Bref e as dimensões do MBI.

**Tabela 1:** Correlação dos domínios do *World Health Organization Quality of Life Assessment* por dimensões do *Maslach Burnout Inventory* de docentes do ensino superior, Manaus, Amazonas, Brasil, 2022, N=140

WHOQOL-Bref		*Maslach Burnout Inventory*		
		Exaustão Emocional	Despersonalização	Realização pessoal
Físico	R	-0,13	-0,04	-0,27
	Valor de *p*	0,114	0,607	0,001
Psicológico	R	-0,22	-0,02	-0,48
	Valor de *p*	0,010	0,858	0,000
Relações sociais	R	-0,10	-0,19	-0,31
	Valor de *p*	0,256	0,024	0,002
Meio ambiente	R	-0,07	-0,24	-0,27
	Valor de *p*	0,420	0,003	0,001

*WHOQOL-Bref - World Health Organization Quality of Life Assessment; R - Coeficiente de Correlação de Spearman.*

Nota-se que a correlação significativa foi encontrada entre a variável “quanto tempo o docente disponibiliza para se dedicar ao trabalho através de atividades fora do horário institucional” (correção de provas/trabalhos, preparação de aulas e orientação) e a dimensão realização pessoal do MBI (p=0,0023).

Salienta-se ainda que a pontuação global do PSQI apontou predominância na classificação do sono ruim (66,4%). Destes, 24 docentes (17,1%) atingiram pontuações que os classificaram com presença de distúrbio do sono. A associação do referido instrumento com o domínio exaustão emocional do MBI apresentou resultados significativos (Tabela 2).

**Tabela 2:** Associação entre o Questionário Índice de Qualidade do Sono de Pittsburgh com a dimensão Exaustão emocional do *Maslach Burnout Inventory* de docentes do ensino superior, Manaus, Amazonas, Brasil, 2022, N=140

Componentes - PSQI	Exaustão Emocional				Valor de *p*
	n (140)	Média (DP)	Mediana	Mínimo-Máximo	
Qualidade subjetiva do sono					
Muito boa	22	32,7 (6,01)	34	19-41	0,005^**^
Boa	70	31,1 (5,78)	31	11-47	
Ruim/Muito ruim	48	34,1 (5,27)	34	22-49	
Distúrbio do sono (vezes/semana)					
1 a 2	99	31,6 (5,61)	32	11-47	0,029^*^
≥ 3	41	34,3 (5,78)	34	22-49	
Uso de medicação para dormir (vezes/semana)					
Nenhuma vez	82	30,9 (5,84)	31	11-46	0,001^**^
< 1	25	34,7 (5,3)	34	28-49	
1 a 2	33	34,4 (4,84)	34	22-44	
Disfunção durante o dia					
Nenhuma	13	32,7 (7,34)	30	26-49	0,005^**^
Pequena	89	31,5 (5,64)	32	11-47	
Moderada/Muita	38	34,4 (5,07)	35	22-44	
Pontuação Global					
Boa	23	30,1 (7,14)	30	11-42	0,007^**^
Ruim	93	32,4 (5,51)	32	15-49	
Presença de distúrbio do sono	24	34,9 (4,35)	35,5	25-44	

PSQI - Questionário Índice de Qualidade do Sono de Pittsburgh;

* Teste de Mann-Whitney;

** Teste de Kruskal-Wallis.

Participantes com qualidade subjetiva do sono (p=0,023) e disfunção durante o dia (p=0,013) classificadas como ruim/muito ruim apresentaram maior escore na dimensão despersonalização do MBI. Docentes com duração do sono < 5 horas manifestaram maior escore na dimensão da realização pessoal (p=0,001). Já indivíduos com presença de distúrbio do sono ≥ 3 vezes por semana, estes apresentaram maior escore na referida dimensão do MBI (p=0,010).

Além do mais, a associação do IDATE estado e traço com a realização pessoal do MBI apresentou resultados significativos (Tabela 3).

**Tabela 3:** Associação entre o Inventário de Ansiedade Traço-Estado com as dimensões do *Maslach Burnout Inventory* de docentes do ensino superior, Manaus, Amazonas, Brasil, 2022, N=140

IDATE	Despersonalização				Valor de *p*
	n	Média (DP)	Mediana	Mínimo-Máximo	
Estado					
Baixo	34	17,6 (3,16)	18	6-24	0,188^*^
Médio	96	17,1 (2,67)	17	10-23	
Alto	10	18,3 (2,63)	18	13-23	
Traço					
Baixo	22	18,7 (2,41)	19	15-24	0,034^*^
Médio	113	17,1 (2,82)	17	6-23	
Alto	5	17,4 (2,07)	17	15-20	
	**Realização pessoal**				
Estado					
Baixo	34	19,4 (5,23)	20,5	6-28	0,006^*^
Médio	96	22,5 (4,53)	23	7-32	
Alto	10	21,1 (3,77)	20,5	13-26	
Traço					
Baixo	22	17,4 (5,58)	16,5	6-27	0,002^*^
Médio	113	22,4 (4,3)	23	7-32	
Alto	5	21,4 (2,97)	21	18-26	

IDATE - Inventário de Ansiedade Traço-Estado;

* Teste de Kruskal-Wallis.

As variáveis intituladas de quantidade de alunos por sala e para orientação apresentaram significância na associação com a dimensão exaustão emocional do MBI (Tabela 4). Docentes com mais de 40 alunos por sala apresentaram maior escore na dimensão despersonalização do que participantes com até 40 alunos por sala (p=0,006). Participantes com mais de cinco anos de instituição possuíam maior escore na referida dimensão (p=0,032). E indivíduos com até dois alunos para orientação apresentaram maior escore na dimensão da realização pessoal (p=0,045).

**Tabela 4:** Associação entre as variáveis laborais pela dimensão exaustão emocional do *Maslach Burnout Inventory* de docentes do ensino superior, Manaus, Amazonas, Brasil, 2022, N=140

	Exaustão Emocional				Valor de *p*
	n	Média (DP)	Mediana	Mínimo-Máximo	
Quantidade de alunos por sala					
Até 40	34	30,62 (5,74)	30,5	15-46	0,017^*^
Mais de 40 alunos	106	33 (5,69)	33	11-49	
Quantidade de alunos para orientação					
Até 2	89	31,82 (5,07)	32	11-46	
2 a 4	23	36 (5,84)	36	27-49	0,028^**^
4 a 6	11	31,18 (5,46)	33	21-38	
Mais de 6	17	31,53 (7,76)	34	15-44	

* Teste de Mann-Whitney;

** Teste de Kruskal-Wallis.

Já a Regressão Linear Múltipla apresentou as variáveis preditoras que melhor explicaram os escores das dimensões do *burnout* (Tabela 5).

**Tabela 5:** Variáveis preditoras das dimensões do *Maslach Burnout Inventory* de docentes do ensino superior, Manaus, Amazonas, Brasil, 2022, N=140

Dimensões	Regressão Linear Múltipla		Valor de *p* ^*^
	Variáveis	Estimativa	
Exaustão emocional	Constante	38,58	<0,001
	2 a 4 alunos para orientação	3,44	0,004
	WHOQOL - Domínio Físico	-0,14	0,001
	Qualidade subjetiva do sono ruim	-2,13	0,022
	Mais de 40 alunos por sala	2,10	0,049
Despersonalização	Constante	16,76	<0,001
	Mais de 40 alunos por sala	1,87	0,003
	Muita disfunção de sono durante o dia	1,42	0,004
	IDATE Traço - Médio	-1,58	0,004
Realização pessoal	Constante	32,94	<0,001
	WHOQOL - Domínio Psicológico	-0,12	0,001
	Elevado tempo de trabalho docente fora da instituição	-0,21	0,002
	Presença de distúrbio do sono na pontuação global	2,02	0,034
	WHOQOL - Domínio Relações sociais	-0,07	0,004
	Uso de medicação para dormir - ≥ 3 vezes por semana	1,72	0,038

* *Nível de significância de 5% (Valor de p < 0,05); WHOQOL - World Health Organization Quality of Life Assessment; IDATE - Inventário de Ansiedade Traço-Estado.*

## DISCUSSÃO

A QV é um foco frequente de estudos científicos, pois seus escores podem atuar como indicadores subjetivos da condição de saúde^(27)^. Os resultados deste estudo indicam que, quanto mais alto o escore no domínio psicológico do WHOQOL-Bref, mais baixo foi o escore na dimensão exaustão emocional do MBI. Além disso, escores mais elevados nos domínios meio ambiente e relações sociais correspondem a menores escores de despersonalização. Esse fenômeno pode ser atribuído às características intrínsecas da profissão, que é influenciada por vários elementos que contribuem para o esgotamento profissional, incluindo, mas não limitado a: excesso de lotação em salas, remuneração insuficiente, falta de segurança e pouca ou nenhuma autonomia^(28-29)^.

Inclusive, quanto maior o escore do domínio meio ambiente, relações sociais, psicológico e físico, menor foi o escore da dimensão da realização pessoal nesta pesquisa. A compreensão da QV é individual, envolvendo parâmetros objetivos/subjetivos correlacionados com inúmeras variáveis e interligadas com a saúde, o lazer, a educação, o ambiente, a segurança, o urbanismo e a tecnologia^(30)^. Na SB, a referida dimensão é classificada de forma reversa, quanto menor o escore, maior é a gravidade. À vista disso, mediante à insatisfação, os docentes podem apresentar condições que prejudicam o seu desempenho, condicionando sentimentos de desesperança, tensão, solidão, fraqueza, preocupação e apatia pelos alunos e colegas de trabalho^(31)^.

Além disso, realizar atividades laborais fora do horário institucional pode predispor a inúmeros prejuízos à saúde emocional do docente^(32)^. Situação que pode resultar no aparecimento de distúrbios do sono, uma vez que somente (20%) dos docentes, neste estudo, relataram dormir mais de sete horas, com latência do sono de até uma hora (43,6%). Condição esta que pode ser explicada pela dinâmica da profissão, marcada por atividades extraclasse, como correção de provas, preparação de aulas, orientações de trabalhos de conclusão de curso e participação em atividades de pesquisa e extensão^(33)^.

Vale ressaltar que a qualidade do sono ruim é descrita como frequente em docentes do ensino superior^(34)^. Outros estudos que utilizaram o mesmo instrumento apresentaram percentuais mais baixos, que favoreceram agravos à saúde biopsicossocial do profissional^(35-36)^. A sobrecarga de trabalho aumentada corrobora para manifestação de distúrbios do sono, condição laboral que explica a qualidade do sono ruim nesta e em outra investigação^(37)^. Docentes com sonolência diurna excessiva podem desenvolver transtornos metabólicos, físicos e endócrinos, predispondo a problemas gástricos e intestinais, déficit no sistema imunológico e distúrbios alimentares, o que afeta a QV^(38)^.

Ademais, estudos ressaltam que o uso de medicação é frequente na classe docente. Uma pesquisa evidenciou que 21,9% dos docentes faziam uso de medicamentos (prescritos ou não pelo médico), como ansiolíticos, hipnóticos, analgésicos e antidepressivos^(33,39)^. Desse modo, a automedicação é realizada na maioria das vezes como forma de aliviar os sintomas desencadeados pelo trabalho. Entretanto, o uso incorreto pode trazer diversas consequências para saúde, com foco na dependência, intoxicação e resistência aos remédios^(40)^.

Docentes que apresentam duração do sono < 5 horas por noite podem apresentar maior declínio cognitivo ao longo do tempo. Tal situação pode ser explicada pela alta exigência no trabalho, possuindo pouco tempo para realizar atividades no âmbito pessoal, desencadeando sinais e sintomas correlacionados com a SB^(41)^. Esses estados laborais interferem na QV e no ambiente de trabalho, apresentando diversos prejuízos no planejamento das aulas. Fator demonstrado como maior risco de adoecimento por estresse ocupacional em outra investigação^(42)^.

De modo similar, a ansiedade está entre os sintomas que mais aparecem nas pesquisas feitas com docentes do ensino superior e afeta os indivíduos mais jovens, com idade inferior a 40 anos, e isso se dá possivelmente por estarem em início de carreira, possuírem pouca experiência e maturidade profissional, o que os torna mais suscetíveis às frustrações laborais^(16)^. Isto corrobora para o aparecimento de sintomas psicossomáticos, como estresse e insônia, Hipertensão Arterial Sistêmica (HAS), além do que, essa situação laboral favorece o desenvolvimento da SB^(43)^.

Já em relação às variáveis sociodemográficas e laborais, estudos evidenciaram que quanto maior o tempo de formação profissional, maior é a avaliação negativa do trabalho^(44-45)^. E ressaltaram que solteiros possuem maior risco de desenvolverem *burnout*, pois dedicam-se, na maioria das vezes, somente ao trabalho. Nesse contexto, a existência de um parceiro afetivo relaciona-se como fator protetor, dando maior segurança, apoio e encorajamento para lidar com os eventos relacionados ao trabalho^(41)^.

Ainda, trabalhar em duas ou mais instituições de ensino pode relacionar-se às condições financeiras, pessoais e laborativas^(46)^. Um estudo internacional realizado no Paquistão demonstrou que 70,3% dos docentes apresentaram moderada exaustão emocional e grave Realização pessoal^(47)^. Esta condição é associada aos problemas de identidade pessoal e à falta de reconhecimento no setor de trabalho, favorecendo o esgotamento profissional^(48)^.

Por fim, a elevada quantidade de alunos por sala de aula aumenta a sobrecarga laboral, pois intensifica o estresse, predispondo a sua cronicidade. Essa condição acarreta prejuízos à saúde psicológica e física dos docentes, predispondo ao aparecimento de dispneia, náuseas, tremores e formigamento nas mãos, taquicardia, baixa produtividade, diminuição do interesse sexual, falta de apetite, sudorese excessiva, mialgia e dificuldade para adormecer^(49)^. Somado a isso, profissionais que estão a mais tempo na instituição de ensino podem apresentar sinais e sintomas correlacionados com a SB, com foco no absenteísmo^(17-18)^.

Com efeito, as variáveis preditoras que se correlacionaram ao esgotamento profissional, neste estudo, necessitam de séria avaliação e análise para que medidas preventivas possam ser instituídas no sentido de impedir o desenvolvimento da SB. Instruir os docentes sobre as estratégias capazes de diminuir as sequelas ocasionadas pela sobrecarga laboral converte-se como uma medida fundamental capaz de apresentar impacto positivo na QV e diminuir as possibilidades de distúrbios relacionados ao sono. Além de que incluir a prática de atividades físicas e de lazer na rotina pode colaborar na diminuição do nível de ansiedade ocasionado pelo ambiente de trabalho, reduzindo as chances do *burnout* ^(50)^.

### Limitações do estudo

A presente pesquisa foi realizada em uma instituição privada de ensino superior, portanto, seus resultados não podem ser generalizados. Contudo, estudos comparativos em instituições públicas e privadas devem ser considerados, objetivando analisar dados semelhantes ou divergentes, frente às diferentes condições laborais.

### Contribuições para a prática

Esta pesquisa minimiza a lacuna na literatura científica sobre a temática abordada, destacando uma série de variáveis modificáveis que corroboram para o desenvolvimento da SB no ambiente de trabalho.

## CONCLUSÕES

No presente estudo, identificou-se diversas variáveis preditoras que favoreceram o esgotamento psicológico na classe docente. Ao considerar a proporção da SB, por meio da regressão linear, evidenciou-se que as seguintes variáveis melhor explicam o seu desenvolvimento: qualidade do sono ruim, mais de 40 alunos por sala, muita disfunção de sono durante o dia, IDATE-Traço, WHOQOL nos domínios Psicológico e Relações sociais, elevado tempo de trabalho docente fora da instituição, presença de distúrbio do sono na pontuação global, uso de medicação para dormir (≥ 3 vezes por semana) e alunos para orientação. Logo, conclui-se que o sistema educacional corrobora para o agravamento deste quadro, uma vez que a dinâmica da profissão é marcada por inúmeras atividades que são realizadas fora do horário institucional, predispondo às crises de ansiedade, alterações na QV e no padrão do sono, gerando agravos à saúde biopsicossocial do profissional.

Diante do exposto, é possível refletir a situação atual dos docentes da instituição privada de ensino superior, em foco, ressaltando a necessidade de alerta no gerenciamento das variáveis preditoras que favoreceram o desgaste físico e emocional dos profissionais avaliados.

Conclui-se que é fundamental elaborar ações de promoção e prevenção da saúde dentro do contexto universitário, para que não ocorra comprometimento da QV, do sono e da saúde dos docentes com consequente comprometimento do ensino e aprendizado dos alunos. Todavia, faz-se necessário novos estudos, ações e programas que promovam a exclusão dessas condições que se correlacionam com o desenvolvimento da SB.
